# The Antennal Pathway of Dragonfly Nymphs, from Sensilla to the Brain

**DOI:** 10.3390/insects11120886

**Published:** 2020-12-16

**Authors:** Silvana Piersanti, Manuela Rebora, Gianandrea Salerno, Sylvia Anton

**Affiliations:** 1 Dipartimento di Chimica, Biologia e Biotecnologie, University of Perugia, 06123 Perugia, Italy; silvana.piersanti@unipg.it (S.P.); manuela.rebora@unipg.it (M.R.); 2 Dipartimento di Scienze Agrarie, Alimentari e Ambientali, University of Perugia, 06123 Perugia, Italy; gianandrea.salerno@unipg.it; 3 IGEPP, INRAE, Institut Agro, Univ Rennes, 49045 Angers, France

**Keywords:** aquatic insects, hemimetabolous, Odonata, ontogenesis, sensory biology, neuroanatomy, morphology, olfaction, mechanoreception, thermo-hygroreception

## Abstract

**Simple Summary:**

The study of the sensory biology in aquatic insects undergoing incomplete metamorphosis, passing from nymphal life in fresh water to adult aerial life, provide great opportunities to understand how Arthropod nervous systems can adapt in response to critical ecological challenges. Here we investigate the antennal sensilla, and the related sensory pathways in the brain, of nymphs of an evolutionarily ancient hemimetabolous aquatic insect, the dragonfly *Libellula depressa*, and compare them with previous data on adults. While antennal sensilla are dramatically different between *L. depressa* nymphs and adults, responding to the need to perceive different cues in water and air, the general morphology of the brain and the sensory circuitry remain quite similar during development. That suggests that the same brain centers are able to process highly diverging information, provided through different sensory structures adapted to water and air. This is in agreement with developmental plasticity that serves as a mechanism to maintain functionality throughout ontogenesis, when the lack of a pupal stage does not allow metamorphic changes of the nervous system. The present data also advance the knowledge on the biology of Odonata, threatened insects in fragile ecosystems, and thus present important results from an evolutionary and conservation biology perspective.

**Abstract:**

Dragonflies are hemimetabolous insects, switching from an aquatic life style as nymphs to aerial life as adults, confronted to different environmental cues. How sensory structures on the antennae and the brain regions processing the incoming information are adapted to the reception of fundamentally different sensory cues has not been investigated in hemimetabolous insects. Here we describe the antennal sensilla, the general brain structure, and the antennal sensory pathways in the last six nymphal instars of *Libellula depressa*, in comparison with earlier published data from adults, using scanning electron microscopy, and antennal receptor neuron and antennal lobe output neuron mass-tracing with tetramethylrhodamin. Brain structure was visualized with an anti-synapsin antibody. Differently from adults, the nymphal antennal flagellum harbors many mechanoreceptive sensilla, one olfactory, and two thermo-hygroreceptive sensilla at all investigated instars. The nymphal brain is very similar to the adult brain throughout development, despite the considerable differences in antennal sensilla and habitat. Like in adults, nymphal brains contain mushroom bodies lacking calyces and small aglomerular antennal lobes. Antennal fibers innervate the antennal lobe similar to adult brains and the gnathal ganglion more prominently than in adults. Similar brain structures are thus used in *L. depressa* nymphs and adults to process diverging sensory information.

## 1. Introduction

Nearly 100,000 species of insects belonging to 12 orders spend one, or more, life stages in freshwater [1]. They encompass pure aquatic insects, such as Odonata, and others, which live in water but retrieve oxygen mainly from air, such as some Coleoptera. Among aquatic insects, we can also distinguish holometabolous species, like mosquitoes, and hemimetabolous species, such as dragonflies, which do not undergo a pupal stage in their life cycle. Considering that all aquatic insects derive from terrestrial ones [1], the successful invasion of lentic and lotic ecosystems required many physiological adaptations, such as new ways to breathe, move, acquire food, but also to sense the environment through the antennae. This can be compared to the earlier land invasion of Hexapoda from a marine ancestor, which required several physiological adaptations to survive outside water, including the evolution of sensory systems [2,3]. Thus, studying the sensory biology of hemimetabolous aquatic insects, passing from fresh water to land by an incomplete metamorphosis, provide an attractive opportunity to understand how Arthropod nervous systems evolve in response to critical ecological challenges.

Despite the fact that insect fauna dominates freshwater aquatic systems, we know very little about their sensory biology and neuroanatomy [4,5]. Among winged insects, Odonata are probably the oldest ones, as testified by the fossil records of the giant Meganisoptera in the late Carboniferous [6], and the indirect evidence that the crown group Odonata have diverged after the late Triassic [7]. Together with Ephemeroptera, they constitute the clade Paleoptera [8,9]. Together with their inability to fold the wings over the abdomen, adult dragonflies and mayflies share big eyes and short, reduced antennae [10]. Dragonfly nymphs live in fresh water, while adults fly over streams, lakes, and small ponds. This implies that the sensory equipment, as well as the nervous system, must change to accommodate the sensory requirements to the profound differences between nymphal and adult habitat [10].

Antennae are the main chemosensory organ in insects, bearing chemoreceptors of different types, sensitivity, and distribution, which play an important role in many behaviors, such as host recognition, mate location, oviposition, aggregation, and defense [11]. Adult Odonata possess thin setaceous antennae bearing only a few olfactory sensilla and thermo-hygroreceptive sensilla along the flagellum [12,13,14]. Differently, mature nymphal dragonflies have bigger antennae that harbor many mechanoreceptive sensilla [10]. These antennae are quite variable in shape [10], but all are equipped with one apical composed sensillum, whose morphology suggests a chemoreceptive function [10,15,16,17,18,19,20]. Other sensilla on the nymphal flagellum are long filiform hairs and short and thick trichoid sensilla, with a mechanoreceptive function, and two subapical coeloconic sensilla [16,18], involved in hygroreception [18,21]. Together with mechanoreception, chemical ecology probably plays a relevant role in the interaction between Odonata nymphs and their environment [22], as suggested by the results of some behavioral investigations [23,24,25,26,27,28,29], and in agreement with the sensory biology of other aquatic insects [4,5].

Antennal sensory receptor neurons require corresponding regions in the brain, processing the incoming information. Whereas development of the brain and especially of sensory neuropil has been well studied in purely terrestrial holometabolous insects, such as *Drosophila melanogaster, Manduca sexta,* and social insects, only one study exists to our knowledge comparing the brain of water-living nymphs and adults, but also here of a holometabolous insect, the mosquito *Aedes aegypti* [30] (and references therein). Brain morphology, and specifically the anatomy of antennal sensory pathways, has been largely ignored in dragonfly nymphs so far [4] and the general anatomy and antennal projections to the brain have been described only in *Libellula depressa* adults [31].

To get insight into the development of the antennal sensory abilities in aquatic hemimetabolous insects, passing from water to air, we describe here the antennal sensilla in the last six nymphal instars of *L. depressa*, together with the general brain structure and the central antennal pathways. We also compare these data with literature available about antennal sensilla in mature nymphs F0 [16,18] and antennal sensilla [13] and brain morphology [31] in adults of the same species. We found that nymphal antennae and brain do not change considerably during development. Moreover, the nymphal brain structure is very similar to the adult brain, despite the considerable differences in antennal sensory equipment and habitat. This might be considered coherent with the gradual metamorphosis of these hemimetabolous insects.

## 2. Materials and Methods

### 2.1. Insects

Nymphs of *L. depressa* Linneus (Odonata, Libellulidae) belonging to the last six nymphal instars, from F5 to F0 [32], were collected in a natural pond in Monte Malbe (Perugia, Central Italy) from winter 2018 to autumn 2019. *L. depressa* performs its development in small standing ponds through 11 post-hatching molts, one from prolarva to F10 and the following ten from F10 to F0 nymphal instars. One additional molt allows F0 mature nymphs to complete the gradual metamorphosis and emerge as winged adults.

The nymphal instar of each insect was determined measuring the total length of the body and the size of the wing pads [32]. In the laboratory, nymphs of each instar were kept in plastic containers with water, detritus, flora, and fauna from the collecting site at 17 ± 2 °C, LD12:12 h light conditions, and were fed ad libitum with plankton.

Only nymphs from F5 to F0 were used for the research, because of technical difficulties connected with small dimensions of earlier instars and, more importantly, because nymphs earlier than F5 are present in the ponds only for few days along two months, July and August [32]. Instars from F5 to F0, on the other hand, stay several months in the ponds, allowing a total development to adults in one (southern Europe) or two years [32].

### 2.2. Scanning Electron Microscopy

To describe sensilla on the flagellum, and in particular to observe the coeloconic apical pegs, antennae were observed under scanning electron microscopy. Dragonfly heads (F0/F5 nymphs) were dissected from anesthetized specimens and fixed for 12 h in 2.5% glutaraldehyde in cacodylate buffer, pH 7.2. For scanning electron microscopy (SEM) analysis, the fixed material, repeatedly rinsed in the same buffer, was then dehydrated by using ethanol gradients, followed by critical-point drying in a critical-point dryer CPD 030 Bal-Tec (Bal-Tec Union Ltd., Balzers, Liechtenstein). Specimens were mounted on stubs with silver conducting paint to expose the antennae from different points of view and allow a complete vision of the sensilla. The stub with the specimens was sputter-coated with gold-palladium in a sputterer Emitech K550X (Emitech, Ashford, England), and observed with a Philips XL30 SEM (Philips, Eindhoven, The Netherlands), at an accelerating voltage of 18 kV.

### 2.3. Neuroanatomy

To reveal the general structure of the nymphal brain, a monoclonal antibody against the *Drosophila* vesicle-associated protein synapsin 1 (SYNORF1, Developmental Studies Hybridoma Bank, University of Iowa, Iowa City, IA, USA) was used. The central antennal sensory pathway was traced in each nymphal stage by Microruby solution (tetramethylrhodamine dextran with biotin, 3000 MW, lysine-fixable, D-7162; Molecular Probes, Eugene, OR, USA) applied with a capillary on the cut flagellum (approximately half the flagellum was cut, bearing the apical composed putative chemoreceptive sensillum and coeloconic sensilla, as well as a large proportion of mechanoreceptive sensilla). In additional F3 nymphs, secondary neurons were traced with Microruby from the antennal lobe (AL) towards the protocerebrum. All anterograde staining preparations were also counterstained with the synapsin antibody to reveal the surrounding structures of the central nervous system.

Insects (F5 to F0 nymphal instars) were immobilized in plastic tubes, adapted to the size of the nymphs, with the head protruding, and the basis of the head fixed with dental wax (Surgident, Heraeus Kulzer Inc., New York, NY USA). The plastic tube was fixed on a microscopy glass slide, the head and antennae of the nymph were dried with tissue and a glass capillary, with the tip adjusted to the size of the antenna and filled with concentrated Microruby in PBS, was slipped over the cut tip of the antenna, and fixed with dental wax on the same slide as the insect. For antennal lobe (AL) anterograde staining, the head capsule was opened, and the AL was perforated several times with a microcapillary containing a Microruby cristal. The head capsule was then covered with the previously removed piece of cuticle. All preparations were kept in a moist chamber in the dark for 5 to 24 h at 18 °C. At the end of the staining period, heads were cut, fixed with an insect pin in a Petri dish filled with dental wax and the cuticle was opened. The opened head was covered with a 4% EM-grade formaldehyde solution (Fisher Scientific, Illkirch, France) in PBS for 1 to 3 h. Subsequently the preparation was rinsed with PBS, the brain was dissected (whenever possible with the gnathal ganglion (GG) attached) and post-fixed overnight in 4% formaldehyde.

For anti-synapsin immunostainings, the nymphal head was directly cut and treated as described for antennal anterograde staining preparations. All brains were then stained as previously described [33]. Briefly, brains were rinsed in PBS, pre-incubated in PBS with 2% NGS and 0.5% Triton X 100, incubated with the anti-synapsin 1 antibody (1:25 in PBS with 0.5% Triton X and 2% NGS for 3 to 5 days). After rinsing, brains were incubated with the secondary antibody (1: 250 in PBS with 1% NGS for 2 to 3 days; Alexa-Fluor-488-conjugated anti mouse IgG; Invitrogen, Thermo Fisher Scientific, France). Brains were finally rinsed, dehydrated in a graded ethanol series, and mounted in methyl salicylate on aluminum slides between two microscopic cover glasses.

Preparations were scanned in a confocal microscope (Nikon A1) with excitation wavelengths of 488 and 561 nm. A PlanFluor objective (10×/NA 0.3) with additional digital zoom was used for image acquisition. Alexa 488 fluorescence was detected at 525 nm fluorescence and Microruby at 595 nm. Stacks of images were acquired at 1024 × 1024 pixels, a 4× frame average and with a stepsize of 2 μm for overview images and 1 μm for detailed scans of the AL and the GG. Stacks were then analyzed and maximum intensity Z-projections of selected sections were produced using Fiji software (Image J, version 2.0.0, National Institutes of Health, Bethesda, ML, USA). The 3D reconstruction of the main brain neuropils was done from optical sections through an anti-synapsin-stained preparation, using Amira 3.1.1 software (Visualization Sciences Group, Mérignac, France).

## 3. Results

### 3.1. Antennal Morphology

**Figure 1:**
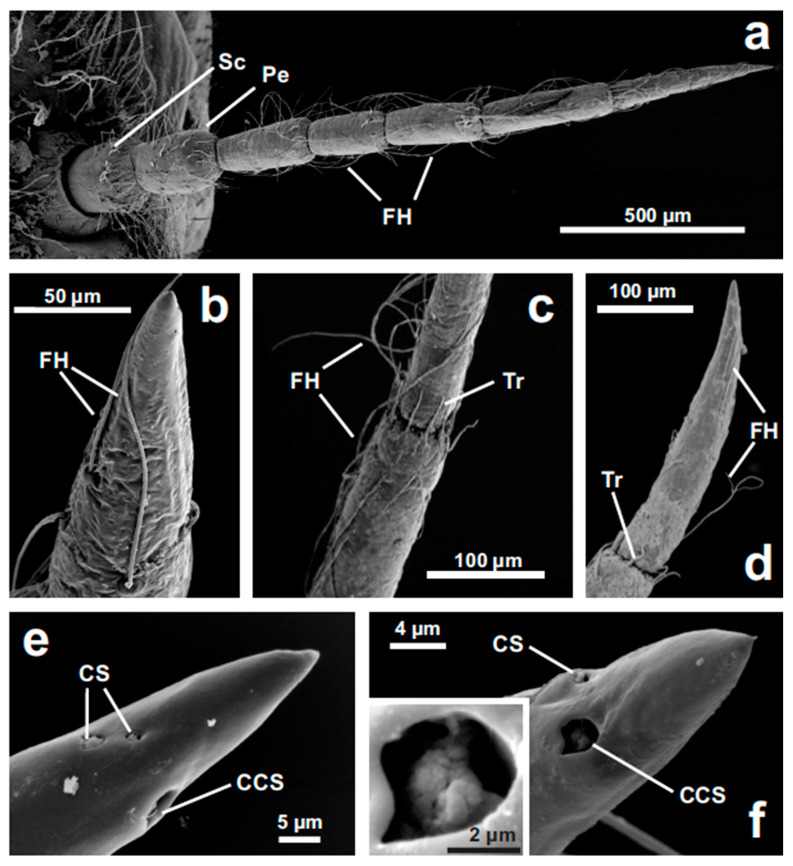
Nymphal (from F-5 to F-0 instars) antenna of *Libellula depressa* under SEM. (**a**) The antenna is composed of scape (Sc), pedicel (Pe) and five flagellar segments, bearing numerous filiform hairs (FH) (F3). (**b**) Filiform hairs (FH) on the last flagellar segment (F3). (**c**) Trichoid sensilla (Tr) in the distal portion of each flagellar segment contact the following segment (F3); FH, filiform hairs. (**d**) Filiform hairs (FH) and trichoid sensilla (Tr) on the distal portion of the antenna (F5). (**e**,**f**) Detail of the composed coeloconic sensillum (CCS) and the paired single coeloconic sensilla (CS) in F4 (**e**) and F5 (**f**) nymphs. The irregular structure of the composed coeloconic sensillum is shown in the inset (**f**).

The antenna of *L. depressa* is very similar in all the investigated instars (F5/F0), except for the dimension, which increases from a total length of 1.55 ± 0.06 mm in F5 to a total length of 3.43 ± 0.08 mm in F0 (*n* = 10 for each instar). The antenna consists of a scape, a pedicel, and a flagellum of five segments (Figure 1a). The flagellum shows many long filiform hairs (Figure 1a–d) and short trichoid sensilla, located in the distal portion of each flagellar segment (Figure 1c,d). Trichoid sensilla do not show any apical pore and have the typical morphology of contact mechanoreceptive sensilla (Figure 1c). The composed coeloconic sensillum is visible at the apex of the antenna in all the investigated instars (Figure 1e,f); its sensory cuticle shows an irregular spongy structure (Figure 1f and inset). The two subapical coeloconic sensilla, characterized by a rough coat, are also present in all the nymphal instars (Figure 1e,f).

### 3.2. General Brain Structure

**Figure 2:**
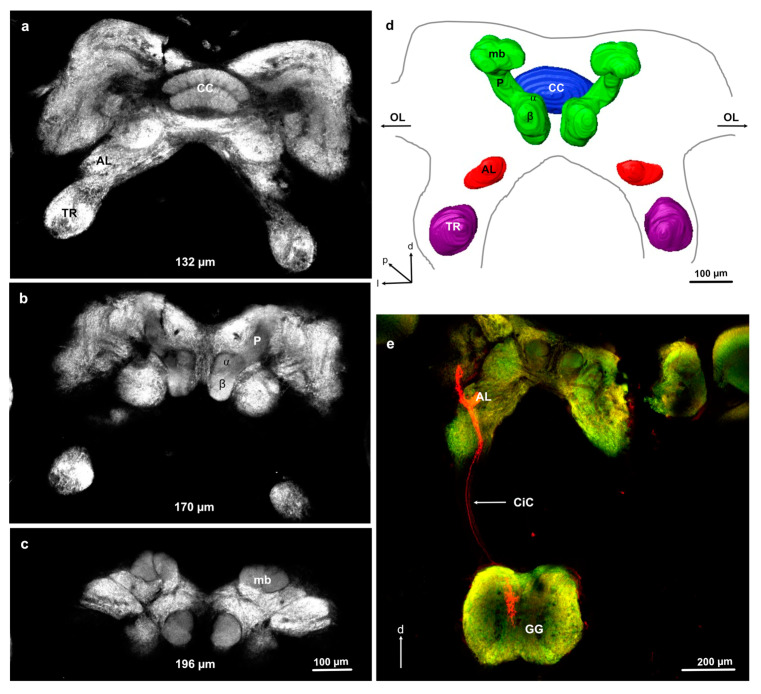
General brain structure and antennal projections in *Libellula depressa* nymph (F3 instar). (**a**–**c**) Frontal optical sections through an anti-synapsin-stained F3 brain at different depths indicated below each image from anterior to posterior. (**d**) 3D reconstruction of the main brain neuropils. (**e**) Maximum z-projection over 38 μm of confocal stack images of antennal axons targeting the antennal lobe (AL) and the gnathal ganglion (GG), illustrating the large distance between brain and GG in nymphs. α alpha lobe of the mushroom body; β beta lobe of the mushroom body; CC central complex; CiC circum-esophageal connective, d dorsal, l lateral; mb posterior part of mushroom bodies, OL arrows indicate where the optic lobes are attached; p posterior; P peduncle of the mushroom body; TR tritocerebrum. Microruby stained axons in red and background staining with the anti-synapsin antibody in green.

**Figure 3:**
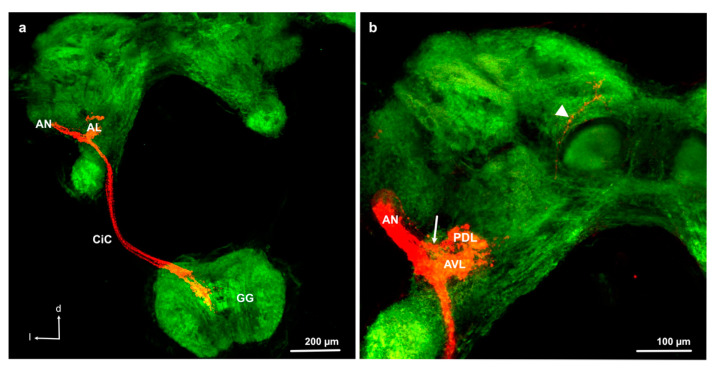
Antennal projections in *Libellula depressa* nymph (F0 instar). (**a**) Antennal nerve (AN) projections to the antennal lobe (AL) and through the circum-esophageal connective (CiC) to the gnathal ganglion (GG). (**b**) Bipartite AN entering the AL (arrow indicating dorsal AN branch), innervating the posterior dorsal (PDL) and anterior ventral (AVL) lobe, and a fiber projecting to the protocerebrum (arrowhead). d dorsal, l lateral. Maximum z-projections over 142 μm (**a**) and 70 μm (**b**) of confocal sections with Microruby stained axons in red and background staining with the anti-synapsin antibody in green.

The nymphal brain in *L. depressa* has the same general structure throughout instars F5 to F0, revealing prominent mushroom bodies, central complex, tritocerebral lobes, and small aglomerular ALs (Figure 2). The mushroom bodies are composed of a pedunculus connected to a medial lobe (β lobe) and a rudimentary vertical lobe (α lobe). The posterior part of the mushroom bodies is slightly enlarged but does not seem to form calyces (Figure 2). The nymphal brain shows very long circumesophageal connectives, creating a large distance ranging from 300 to 500 μm in the investigated instars (determined from 1 intact preparation in each investigated instar) between the brain and the GG, which is located ventrally (Figure 2e and Figure 3a).

### 3.3. Antennal Projections to the Brain and GG

**Figure 4:**
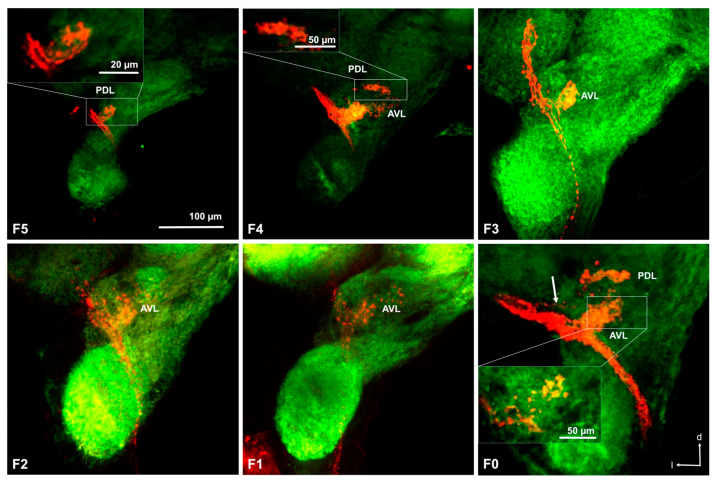
Antennal lobe projections in the 6 investigated nymphal instars of *Libellula depressa* (F5/F0 instars). Note that all maximum z-projections of stacks of optical sections (over 40 μm in F5, 32 μm in F4, 14 μm in F3, 21 μm, 28 μm in in F1 and F0) were taken at the same scale. Insets show a higher magnification of individual optical sections from the respective antennal lobe area. AVL anterior-ventral part of the antennal lobe, PDL posterior dorsal part of the antennal lobe (see [31]). The arrow indicates the weakly stained upper antennal nerve branch innervating the PDL area. d dorsal, l lateral. Microruby stained axons in red and background staining with the anti-synapsin antibody in green.

Anterograde staining from the cut flagellum revealed the structure of the antennal sensory pathways in nymphs (Figure 2e and Figure 3). We obtained 5, 4, 4, 1, 6, and 5 successful stainings in F0 to F5 nymphs, respectively. As can be seen in Figure 4, the size of the AL increases with nymphal instars from less than 50 μm in diameter in F-5 to almost 100 μm in F0, even though precise quantitative analysis of the AL volume was not possible due to high variability of branching of the stained axons within the ALs and impossible precise delimitation of AL boarders in anti-synapsin stained preparations. In all examined nymphal instars, the antennal nerve enters the AL from an anterior–lateral direction and axon branches can be seen within one or two regions within the AL, depending on the preparations. We named the regions anterior ventral part (AVL) and posterior dorsal part (PDL) of the AL, as defined earlier in adult *L. depressa* brains [31]. In all instars we obtained preparations with branches in the AVL alone, in F0, F4, and F5 preparations we found stainings in both areas and in two F5 preparations we found branches only in the PDL area. In the PDL area, a protoglomerular organization could be seen in some preparations (Figure 4 F5, F4, F0), whereas no glomerular structure was seen in the AVL area of the AL (Figure 4). In the last nymphal stage F0, we could identify two antennal nerve branches entering the antennal lobe (Figure 3b), and few projections to the medial protocerebrum (Figure 3b). In earlier nymphal instars, no separate branches of the antennal nerve, or fibers projecting to the protocerebrum, could be clearly identified (Figure 4).

**Figure 5:**
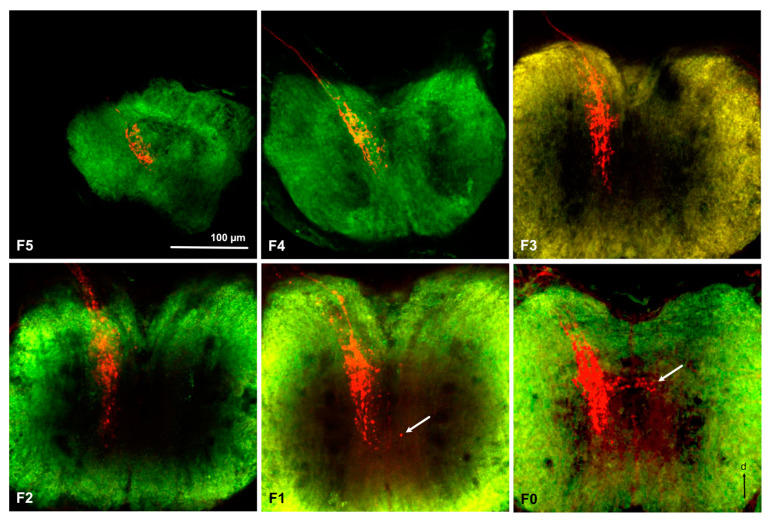
Projections to the gnathal ganglion in the 6 investigated nymphal instars of *Libellula depressa* (F5/F0 instars). Note that all maximum z-projections of stacks of optical sections (over 14 μm in F5, 26 μm in F4, 11 μm in F3, 14 μm, 17 μm in in F1, and 26 μm in F0) were taken at the same scale. Arrow indicates contralateral branches of the axons in the F0 and F1 instars. d dorsal. Microruby stained axons in red and background staining with the anti-synapsin antibody in green.

Prominent fibers from the antennal nerve, passing the AL posterior-laterally, project to the gnathal ganglion (Figure 2e, Figure 3, and Figure 5). They innervate the same ipsilateral portion of the central part of the GG in all examined nymphal instars (Figure 5). Only rarely we found a few fibers crossing the midline to the contralateral side in F1 and F0 instars (Figure 5). As for ALs, an overall increase in size of the GG during development can be observed in Figure 5, from about 200 μm width in F5 to more than 500 μm in F0 nymphs and adults.

### 3.4. Projections from the Antennal Lobe to the Protocerebrum

**Figure 6:**
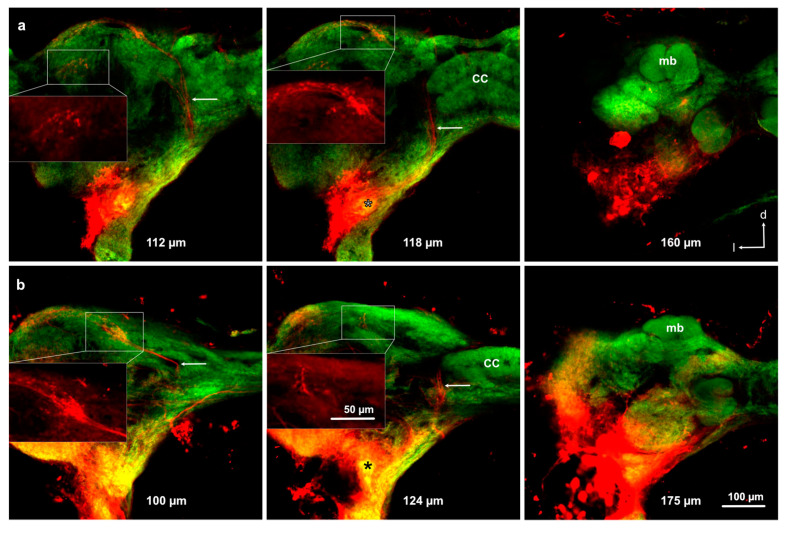
Projection neurons originating from the antennal lobe in *Libellula depressa* nymphs (F3 instar) in two individuals (**a**,**b**). Individual sections from anterior (left) to posterior (right). Distance from anterior side of the brain is provided in micrometers. Asterisks indicate dye injection site into the antennal lobe. Arrows indicate medial antennal lobe tract. Insets show z-projections over 10 μm of the Microruby-stained axons (the same scale bar applies for all insets). cc central complex, d dorsal, l lateral, mb frontal part of mushroom bodies. Note that central neurons project through the medial antennal lobe tract to the lateral protocerebrum, but not to the mushroom bodies. Microruby stained axons in red and background staining with the anti-synapsin antibody in green.

Five successful preparations of anterograde stainings from the ALs revealed neurons projecting to different areas within the protocerebrum, but not to the mushroom body (Figure 6). No branches of antennal lobe projection neurons were found in the posterior-dorsal part of the mushroom bodies, indicating that this part does not represent mushroom body calyces (Figure 6).

## 4. Discussions

### 4.1. Sensory Equipment of L. depressa Antennae during Ontogenesis

The antennal sensilla of *L. depressa* nymphs do not show any relevant change during the development. Nymphs perform 10 molts from F10 to F0 instars, spending one or two years in small shallow ponds, rich of vegetation and characterized by mud and turbid water [32]. During this period, insects stay partially embedded into the bottom debris and catch small aquatic preys using their modified labium, called mask, with an ambush “sit and wait” strategy [22]. As previously described in mature nymphs F0 [16], all nymphal instars show a flagellum covered by long filiform hairs, able to respond to faint movements of the surrounding medium because of their thin seta inserted in a complex socket [16,34]. Filiform hairs have been reported in all Odonata nymphs investigated so far, particularly numerous in species living in lentic water environments [10,19], where hairs can be easily used to perceive preys and predators [35]. In the distal portion of each flagellar segment we found a crown of trichoid sensilla without pores, characterized by the typical morphology of contact mechanoreceptive sensilla [10,34]. Because of their position, these sensilla are probably able to perceive movements of flagellomeres, one in relation to the other, working as proprioceptors.

At the apex of the antenna all the nymphal instars show one composed coeloconic sensillum with a spongy appearance, which is the best candidate for a chemoreceptive role [10]. As in other dragonfly species [17,19], this sensillum is innervated by two groups of three neurons entering the peg without dendrite sheath [16,18]. The sensory peg shows an irregular coat where true pores are not visible [10,16,18]. The absence of pores does not preclude chemosensory function in water, where eluted chemicals could simply diffuse through the cuticle, similarly to what was observed in the aesthetascs of decapods [36,37]. The presence of chemoreceptors at the apex of the antennae in nymphal dragonflies is in agreement with some evidence of chemically mediated food detection by the antennae [38], together with behavioral investigations that report the ability of dragonfly nymphs to respond to chemicals produced by insectivorous fish, or conspecifics alarmed by these predators [23,24,26,28,39,40].

Two additional coeloconic pegs are always visible on the subapical portion of the antenna. They have a rough coat in close contact with a thick dendrite sheath, all tightly adherent to the enwrapped sensory neurons [18]. This morphology suggests a thermo-hygroreceptive function [41], supported by behavioral [21,42] and electrophysiological results [18].

Despite the incomplete metamorphosis of dragonflies, the sensory equipment of the nymphs is completely different from the one reported in the adult [12,42,43], obviously reflecting adaptation associated with different ecological niches and life strategies. For example, both adults and nymphs are predators, but nymphs catch freshwater macroinvertebrates in dark environments with their mask, and entrust prey perception mainly to mechanoreceptors [22,35]; differently adults chase in full light, catching flying insects that are mainly detected by the eyes [22]. Both nymphs and adults can perceive chemical cues from prey, such as volatile compounds, probably acids and amines, for adults [44], and amino acids, as proline and glycine, eluted in water for nymphs [38]. Further, considering mechanoreception, the higher density of water with respect to air allows vibrations of the medium to be carried much further in fresh water than in the atmosphere, while vision underwater can be very challenging, particularly in turbid and dark ponds. This explains why *L. depressa* nymphs have small eyes and use the numerous filiform hairs as the main organ for prey detection [35]. In air, the thin and smooth antennal flagellum of adults, which never contacts substrates, does not show any mechanoreceptive sensilla (except for some rare campaniform sensilla) [10,13], but is used itself as a bristle, connected with the Johnston’s organ in the pedicel to inform insects about speed and direction of the flight [45,46]. On the latero-ventral side of the smooth flagellum, both male and female adults have some porous coeloconic pegs (around 40 ± 10 in *L. depressa*) and deeply sunken sensilla styloconica (between 80 and 120 in *L. depressa*), without pores, an irregular cone, and a close connection between the sensory cuticle and the dendrites [13,47]. More precisely, multiporous coeloconic pegs are involved in the perception of amines and carboxylic acids [13,44,48], and are related with the ability of dragonflies to move towards prey [49], mates [50], and oviposition site odors [51]. Deeply sunken sensilla styloconica, differently, are related to the perception of humidity, temperature, and CO_2_, demonstrated by electrophysiological recordings [48,52]. The dominant role of vision in the biology of adult dragonflies [22] makes it very difficult to investigate the role of other senses in the field [53] (Piersanti et al., unpublished personal data). Otherwise, we can suppose that adult thermo-hygroreceptors play a role in weather-associated behaviors, such as male’s territoriality depending on sun exposure, taxis behavior with respect to water or sun-basking for thermoregulation [52]. Differently, in nymphs, thermo-hygroreceptors are able to drive insects to moist refuges when exposed to dehydration [21,42], such as in drying ponds during hot summers.

### 4.2. Sensory Antennal Pathways in the Central Brain of L. depressa during Ontogenesis

Coherently with the antennal sensory equipment and nymphal ecology, the brain morphology and sensory pathway do not change profoundly between the different examined nymphal instars. In spite of clear differences in antennal sensilla equipment, differences between the nymphal and adult brain structure were less pronounced. The principal brain structures are similar in dragonfly adults and nymphs, mainly the size of the system changes. Nymphs possess longer connectives of several hundreds of μm between the supra-and sub-esophageal parts of the CNS than adults, which have a more compact CNS and connectives of less than 100 μm between the tritocerebral lobes and the GG [31]. In both nymphs and adults we found prominent mushroom bodies without calyces, an evident central complex, small aglomerular ALs and visible tritocerebral lobes, connecting to the GG through the circum-esophageal connectives. Nymphs possess mushroom bodies with reduced α lobes compared to adults [31].

The differences in antennal projections of F0 nymph compared to other nymphal instars resemble what has been found in adults: (i) partitioning of the antennal nerve in two branches, (ii) few long fibers of the antennal nerve projecting to the protocerebrum, and (iii) the presence of some fibers crossing the midline to the contralateral side in the GG. However, a partitioned antennal nerve might also be present in other instars because we only obtained relatively few and rather variable successful stainings. In adult *L. depressa*, Rebora et al. [31] observed a bipartite antennal nerve and two corresponding deutocerebral projection areas and hypothesized that one branch carries olfactory sensory neurons, while the other carries the axons of thermo-hygroreceptive neurons innervating a separate area. We suggest that the presence of a second branch in F0 nymphs (Figure 5), although less visible than in adults, could be related with the fact that mature nymphs are partially adapted to the aerial environment and can survive in absence of water [21,42] using the mesothoracic spiracles to breath air some days before emergence [22,54]. In this terrestrial condition, mature nymphs probably rely on the antennal thermo-hygroreceptors to remain in a suitable environment [42]. If the two deutocerebral projection areas identified in some nymphal preparations correspond to the two parts of the AL in adults, potentially receiving separate input from olfactory and thermo-hygroreceptive neurons respectively [31], or if they correspond to areas receiving olfactory and mechanosensory input separately, needs to be investigated with single sensillum anterograde staining in the future. Because of the major role of mechanoreception in the immature aquatic stage [10,22], it is an important next step to be investigated. Typically, insect antennal mechanoreceptive neurons send axonal projections to the GG and the antennal mechanosensory and motor center (AMMC) situated in the deutocerebrum [34]. We assume that mechanosensory fibers projecting to the GG in nymphs come from the numerous antennal mechanoreceptive neurons, while the adult flagellum is practically devoid of mechanoreceptive sensilla except for some campaniform sensilla [13]. If the AMMC in nymphs is situated very closely to the AL, it might be difficult to differentiate it from the AL. Both nymphal and adult ALs are aglomerular, but in the adult AL spherical knots have been described as protoglomerular structures [31], which we could also identify in the posterior dorsal lobe of the AL in nymphs.

Axons ascending to the protocerebrum, stained in our antennal anterograde stainings in mature nymphs have been observed also in adult *L. depressa*, and were interpreted as long olfactory fibers [31] directly carrying olfactory inputs to the protocerebrum [55,56]. Similar ascending fibers bypassing the AL and extending to the protocerebrum have been reported also in ancient Archaeognatha [57] and other insect species [2,58], but if they originate indeed from the antenna or if they represent transsynaptically stained projection neuron fibers is unknown so far. In locusts similar projection patterns have been observed earlier in axons originating from internal mechanoreceptive neurons from the legs, such as chordotonal organs or stretch receptors [59], but we do not know if dragonfly nymphal antenna contain such receptors.

In both nymphs and adults, central processing of olfactory cues seems not to require calyces of the mushroom bodies, but other areas of the protocerebrum seem to function as secondary olfactory centers. This has been confirmed in the nymphal stage by anterograde staining of the AL tracts with Microruby, which shows neurons projecting to the lateral protocerebrum but not to the mushroom bodies. A similar pattern was observed in *Ischnura elegans* Vander Linden (Odonata, Coenagrionidae) adults, investigated with the same technique (Piersanti, personal observations). Our findings are in line with the absence of calyces in the Odonata brain reported earlier [60].

The similarity in brain organization between different nymphal instars and adult dragonflies is in agreement with developmental strategies of hemimetabolous insects. Whereas holometabolous insects undergo neurometamorphosis by means of postembryonic neurogenesis, programmed cell death, and reorganization of the nymphal neural circuits in the transition from behaviorally “simple” nymphs to more complex adults, hemimetabolous insects maintain neuropil functionality throughout the entire development [61,62]. Developmental plasticity can contribute to a fully functional brain during nymphal development, as demonstrated for the mushroom bodies of terrestrial hemimetabolous insects, such as cockroaches [62].

Evidence of olfactory-guided behavior in both dragonfly nymphs and adults [10,38] confirm, however, that neither well-defined glomeruli in the AL, nor calyces of the mushroom body are required for a functional olfactory system, as indicated earlier in Hemiptera [63].

## 5. Conclusions

Odonata nymphs conserve the same antennal sensory structures during development but show strong differences in antennal sensory equipment with respect to adults. Interestingly, the brain and sensory pathways do not show dramatical changes at our scale of investigation between nymphs and adults. Thus, in adult and nymphal dragonflies, the same brain regions seem to be able to process highly diverging information provided through different sensory structures, in air and water, respectively. We might here be confronted with central nervous structures that adapt through minimal tuning to new requirements [64].

Hemimetabolous aquatic insects, namely Odonata, Ephemeroptera and Plecoptera, must maintain the full functionality of antennal sensilla and brain circuitry during the aquatic development because they do not have a pupal stage, but when they emerge from the water as winged adults, they need to completely reorganize their sensory abilities to adapt to the new aerial environment [4]. Not only dragonflies show profound differences between nymphal and adult antennae, but other aquatic insects also drastically change morphology and function of the antenna from water to air (see review in [10]), such as mayflies [65], stoneflies [66,67], and mosquitoes [68,69]. Very few data are available on the neuroanatomy of purely aquatic insects, and no investigations have been performed on the development of the nervous system (see review in [4]), except for holometabolous mosquitoes, due to their evident importance for disease transmission in humans [30].

In this context, our description of the antennal sensilla, the related sensory pathways in the brain of *L. depressa*, and the comparison of these results with data available for adults allow for the first time to trace relevant aspects of brain development in hemimetabolous aquatic insects, characterized by an incomplete metamorphosis able to guarantee the passage from an aquatic nymph to a winged adult. In addition, the present data reinforce the knowledge on the biology of Odonata, threatened insects in fragile ecosystems, such as inland waters. These results are particularly interesting from an evolutionary and conservation biology perspective. We cannot ignore that the diversity of insect species and evolutionary strategies provide a strong benefit for the study of neurobiology, also in consideration of the shared principles of brain development and organization of sensory systems in insects and vertebrates [70,71,72].
